# Induction of foci of altered, γ-glutamyltranspeptidase-positive hepatocytes in carcinogen-treated rats fed a choline-deficient diet

**DOI:** 10.1038/bjc.1979.176

**Published:** 1979-08

**Authors:** M. A. Sells, S. L. Katyal, S. Sell, H. Shinozuka, B. Lombardi

## Abstract

**Images:**


*Br. J. Cancer* (1979) **40**, 274

### INDUCTION OF FOCI OF ALTERED, γ-GLUTAMYLTRANSPEPTIDASE-POSITIVE HEPATOCYTES IN CARCINOGEN-TREATED RATS FED A CHOLINE-DEFICIENT DIET

M. A. SELLS*, S. L. KATYAL*, S. SELL†, H. SHINOZUKA*, AND B. LOMBARDI*

*From the *Department of Pathology, University of Pittsburgh School of Medicine, Pittsburgh, PA* 15261, *and †Department of Pathology, University of California, San Diego, La Jolla, CA* 92093

Received 7 February 1979 Accepted 10 April 1979

**Summary.—A series of experiments was performed to investigate whether, after exposure of rats to a chemical hepatocarcinogen, feeding a choline-deficient (CD) diet would promote the proliferation of initiated liver cells, and their evolution to foci of altered γ-glutamyltranspeptidase (GGT)-positive hepatocytes, without subjecting the animals to further experimental manipulations.**

**Diethylnitrosamine (DEN), in single doses of 15–150 mg/kg body weight, was injected into male, Sprague–Dawley rats, either intact or 18 h after a partial hepatectomy (PH). The animals were then fed either a CD or a choline-supplemented (CS) diet for 2–8 weeks. Emergence in the liver of foci of altered, GGT+ hepatocytes was studied by histological and histochemical techniques. Foci, in varying numbers, developed in the liver of all rats fed the CD diet. The number of foci induced was larger when DEN was administered after PH rather than to intact rats. Foci developed in none of the livers of rats fed the CS diet, except in one experiment in which 30 mg DEN/kg body weight was injected after a PH. In all cases, foci of altered, GGT+ hepatocytes were shown to be α-foetoprotein after immunofluorescence staining of liver sections.**

**It is concluded that feeding a CD diet exerts a strong promoting action on the proliferation and further evolution of liver cells initiated by a chemical carcinogen, providing the basis for a new and efficient procedure for the induction of foci of altered hepatocytes in rat liver.**

It has been shown conclusively in recent years that the process of chemical carcinogenesis in the liver consists of at least 2 basic, probably multi-step, stages: initiation and promotion (Peraino *et al.*, 1973; Pitot *et al.*, 1978). The initial interaction of the carcinogen with target cells generates a small population of initiated cells with the acquired property or properties of evolving eventually into neoplastic cells. Establishment of the latter, however, depends upon the intervention of factor(s) which promote specifically the proliferation and further evolution of initiated cells. In the case of hepatocarcinoma induction, a sequential emergence in the liver occurs, as a consequence of initiation and promotion, of successfully smaller populations of hepatocytes displaying phenotypic changes, through which the entire process evolves from normality to preneoplastic lesions and malignancy (Farber, 1976). Among the induced phenotypic alterations are changes in enzyme activities so characteristic as to represent reliable histochemical markers for the identification of the newly emerging populations of liver cells. Thus, islands of hepatocytes deficient in ATPase and/or glucose-6-phosphatase activities have been shown to appear within a short time after exposure of experimental animals to several chemical hepatocarcinogens (Scherer *et al.*, 1972). Scherer & Emmelot (1975, 1976) have devised an efficient and reproducible procedure for the induction of these islands, and have used it to study and define several of their properties. In the procedure, a single dose of diethylnitrosamine (DEN) a potent hepatocarcinogen, is administered to rats 20 h after partial hepatectomy (PH). Within 8 weeks, numerous microscopic islands of altered hepatocytes are formed which, after histochemical staining, can be easily identified, quantitated and sized. The number and size of the islands have been shown to be proportional to the dose of DEN administered, the enzyme deficiencies to persist in the island cells for many months, and the islands to be associated with the ultimate appearance of hepatocellular carcinomas, even though they can be induced also by single, low, non-carcinogenic doses of DEN.

More recently, a different procedure has been described by Solt & Farber (1976). In this, a single, large dose of DEN (or of other chemical carcinogens) is administered to intact rats in order to initiate liver cells. Proliferation of the latter is then selectively stimulated by application of a "selection pressure" consisting of 2 components, PH, and administration of 2-acetylaminofluorene (AAF). The "selection pressure" is predicated on the fact that initiated liver cells also acquire resistance to the cytotoxic and cytostatic effects of chemical carcinogens such as AAF, whilst uninitiated cells remain sensitive to those effects (Solt *et al.*, 1977). Thus, the intense and generalized stimulus for cell growth provided by PH is applied only to initiated cells. Indeed, within 10 days after PH, numerous foci and minute but visible nodules of altered hepatocytes are formed in rats previously injected with DEN. The rapidity with which the foci emerge, and the size they attain, is most probably due to the fact that Solt & Farber's procedure evokes not only the steps of the initiation stage of chemical hepatocarcinogenesis, but also stimulates or heightens early steps of the promotion stage. The elicited foci and nodules show a high activity of the enzyme γ-glutamyl-transpeptidase (E.C.2.3.7.2, GGT) in contrast to normal hepatocytes and hepatocytes surrounding the foci and nodules, which express no histochemical activity of the enzyme. Identification, quantitation and sizing of the foci and minute nodules can thus be performed easily after histochemical staining of GGT. Even though the procedure suffers from some shortcomings, primarily due to the use of 2 different carcinogens, it has already proved to be a very useful tool in the analysis of the early steps and cellular events of chemical hepatocarcinogenesis (Cayama *et al.*, 1978; Laishes & Farber, 1978).

Feeding a choline-deficient (CD) diet to rats markedly enhances hepatoma induction by chemical carcinogens (Lombardi & Shinozuka, 1978; Shinozuka *et al.*, 1978*a*, *b*). In a previous study (Shinozuka *et al.*, 1979) we used Solt & Farber's procedure to investigate whether the CD diet modifies the initiation stage and/or the promotion stage of liver carcinogenesis. Whereas the diet appeared to have no effect on initiation, evidence was obtained that it has a strong promoting action. Indeed, after administration of a single initiator dose of DEN, a selection pressure consisting of feeding a CD diet containing AAF was found to promote the proliferation of initiated cells, and their evolution to foci or altered hepatocytes, as effectively as the selective pressure used by Solt & Farber (1976). In other words, the diet could effectively replace PH as one of the selection pressures. In this paper we report the results of further studies showing that feeding a CD diet alone, without any other manipulation of the animals, can efficiently promote the proliferation of initiated liver cells and their evolution to foci of altered hepatocytes.

### MATERIALS AND METHODS

*General and analytical procedures.*—Male Sprague–Dawley rats (Zivic Miller Laboratories, Allison, Park, PA) with an initial body weight of 150–200 g were used. The animals were housed individually in metal cages in a room with temperature, humidity and light controls. Food and water were given *ad libitum*. The animals were maintained on laboratory chow (Purina, Ralston Purina Co., St Louis, MO) for at least one week before the beginning of the experiments. Semisynthetic, semi-purified CD and choline-supplemented (CS) diets were prepared as previously described (Shinozuka *et al.*, 1978*c*). DEN (Aldrich Chemicals, Madison, WI) was dissolved in a 0·9% NaCl solution and was administered i.p. PHs were performed according to the method of Higgins & Anderson (1931). The animals were fasted overnight before being killed, and anaesthetized with pentobarbital (60 mg/kg body wt). A blood sample was withdrawn from the abdominal aorta, and the liver was resected and weighed. Serum aspartic amino-transferase (E.C.2.6.1.1, SGOT), plasma GGT and α-foetoprotein (AFP) and liver AFP were determined as previously indicated (Shinozuka *et al.*, 1978*c*; Sell & Gord, 1973). Blocks of liver tissue were fixed in Stieve's solution, and sections were stained with haematoxylin-eosin (HE) for light-microscopic examination. Histochemical localization of GGT was performed by the method of Rutenberg *et al.* (1969). Blocks of liver were quickly frozen in dry ice and 5μm cryostat sections were cut and fixed in acetone. The sections were incubated in freshly prepared medium containing γ-glutamyl-4-methoxy-2-naphthylamide as substrate, and fast blue-BBN as coupling agents. The sections were counterstained with haematoxylin. Control sections incubated in a similar medium without substrate were uniformly negative. All reagents were obtained from Sigma Chemical Corp., St Louis, MO. Foci of $GGT^+$ hepatocytes with a diameter larger than 125 μm were counted, and their number per $cm^2$ of sections determined. The surface area of the sections was measured with the aid of a planimeter, and the size of the foci with the aid of an AO-ocular micrometer. The number of foci per liver was calculated as indicated by Scherer *et al.* (1972). Immunofluorescence staining of liver sections for AFP and albumin was performed as previously described (Sell, 1978). Differences between the means were evaluated statistically by Student's *t* test and regarded as significant if $P < 0{\cdot}05$.

*Experimental designs.*—Five experiments were performed, and the basic design of the experiments is presented diagrammatically in Fig. 1. Experiments 1–4 were designed to test whether feeding the CD diet alone, without subjecting the animals to other manipulations or feeding of AAF, could promote the proliferation of initiated cells, and their evolution to foci of altered, $GGT^+$ hepatocytes. DEN, 150 or 30 mg/kg body wt, was administered to intact animals in Expts 1 and 2, respectively. In Expts 3 and 4, 30 mg or 15 mg DEN/kg of body weight, respectively, were administered 18 h after PH. Then, all animals were fed laboratory chow for 1 week, in order to allow recovery of the liver from any toxic effect of the carcinogen. At this point, the animals in each experiment were divided into 2 groups, one of which was fed a plain CS diet, and the other a plain CD diet. Subgroups of 3–4 rats were killed after 2–8 weeks of feeding the CS or CD diet.

Expt 5 was designed to test whether the CD diet could substitute for AAF as a selective agent in Solt and Farber's procedure, in which PH follows DEN exposure. DEN, 30 mg/kg body wt, was administered to intact rats. After being fed laboratory chow for 1 week, the animals were divided into 2 groups, one of which was fed a plain CS diet, and the other a plain CD diet. One week later, PH was performed on all animals, and 3–4 rats were killed at the end of the 2nd and 4th week of feeding the CS or CD diet.

### RESULTS

The results of all experiments are shown in Table I. Within each experiment there was no statistically significant difference in body weight of rats fed the CS or CD diet. However, the liver weight of rats fed the CD diet was consistently about double that of rats fed the CS diet. This finding is most probably accounted for by the accumulation of fat that occurs in the liver of rats on a CD diet (Lombardi, 1971).

### *Histopathology*

In all experiments, 3 rats were killed 1 week after injection of DEN. The liver of these animals showed no evidence of necrosis, inflammation, or fibrosis. A small number of extramedullary haemopoietic foci was present in the liver of rats injected with 150 mg DEN/kg (Expt 1). In rats subjected to PH (Expts 3 and 4), regeneration of the organ was fairly complete by 1 week.

TABLE I.—*Body and liver weights, number and diameter of foci of GGT⁺ hepatocytes*

| Diet* | Weeks on diet | No. rats | Body wt (g) | Liver wt (g) | Foci/cm² section | Diameter (μm) | Foci/liver × 10⁻³ |
|---|---|---|---|---|---|---|---|
| Expt 1 (IR—150 mg DEN)† | | | | | | | |
| CS | 2 | 3 | 285 ± 15 | 8·7 ± 0·5 | — | 0 | 0 |
| CD | 2 | 3 | 297 ± 9 | 20·1 ± 0·8 | 1·2 ± 0·6 | 150 ± 10 | 2·9 ± 1·7 |
| CS | 3 | 3 | 307 ± 19 | 8·9 ± 1·0 | — | 0 | 0 |
| CD | 3 | 3 | 344 ± 5 | 24·3 ± 1·3 | 2·8 ± 1·4 | 192 ± 20 | 4·9 ± 2·0 |
| Expt 2 (IR—30 mg DEN) | | | | | | | |
| CS | 4 | 3 | 320 ± 28 | 9·3 ± 1·2 | — | 0 | 0 |
| CD | 4 | 3 | 312 ± 45 | 21·4 ± 2·8 | 4·6 ± 0·4 | 172 ± 6 | 6·9 ± 0·5 |
| Expt 3 (PH—30 mg DEN) | | | | | | | |
| CS | 2 | 4 | 240 ± 11 | 6·8 ± 0·5 | 1·0 ± 0·7 | 158 ± 8 | 0·5 ± 0·3 |
| CD | 2 | 4 | 259 ± 5 | 15·7 ± 1·3 | 8·6 ± 0·8 | 189 ± 13 | 7·8 ± 0·5 |
| CS | 4 | 4 | 367 ± 28 | 10·4 ± 1·3 | 0·9 ± 0·8 | 150 ± 10 | 1·0 ± 0·1 |
| CD | 4 | 4 | 316 ± 16 | 19·9 ± 1·7 | 27·3 ± 3·9 | 216 ± 22 | 25·0 ± 3·0 |
| Expt 4 (PH—15 mg DEN) | | | | | | | |
| CS | 4 | 3 | 423 ± 32 | 12·8 ± 1·7 | — | 0 | 0 |
| CD | 4 | 3 | 383 ± 23 | 23·3 ± 2·4 | 18·0 ± 6·9 | 203 ± 27 | 14·3 ± 4·1 |
| CS | 8 | 3 | 543 ± 18 | 15·9 ± 5·4 | — | 0 | 0 |
| CD | 8 | 3 | 500 ± 29 | 24·8 ± 1·4 | 21·7 ± 0·9 | 259 ± 19 | 16·2 ± 2·5 |
| Expt 5 (IR—30 mg DEN—PH) | | | | | | | |
| CS | 2 | 4 | 236 ± 11 | 6·6 ± 0·5 | — | 0 | 0 |
| CD | 2 | 4 | 229 ± 10 | 11·3 ± 0·6 | 1·5 ± 0·9 | 182 ± 6 | 1·0 ± 0·6 |
| CS | 4 | 4 | 329 ± 12 | 8·9 ± 0·5 | — | 0 | 0 |
| CD | 4 | 4 | 307 ± 12 | 18·3 ± 0·9 | 9·4 ± 0·6 | 269 ± 30 | 6·8 ± 0·8 |

Each value represents the mean ± s.e.
* CS, choline-supplemented; CD, choline-deficient.
† Diethylnitrosamine (DEN) administered to intact rats (IR), or 18 h after partial hepatectomy (PH).

FIG. 1.—Diagrammatic representation of the basic design of the experiments. DEN, diethylnitrosamine; PH, partial hepatectomy; LC, laboratory chow; CS, choline-supplemented diet; and CD, choline-deficient diet. For other details, see text.

FIG. 2.—Foci of altered, basophilic hepatocytes (arrows) in the liver of a rat injected with 30 mg DEN/kg body wt 18 h after a partial hepatectomy, and killed after 2 weeks of feeding a choline-deficient diet. Note how the hepatocytes in the foci are only slightly fatty, in contrast to the extensive fatty infiltration of the surrounding parenchyma. H. and E. × 30.

FIG. 3.—Higher magnification of a focus of altered, basophilic hepatocytes in Fig. 2. Note mitosis of hepatocytes. H. and E. × 150.

Within 1 week of feeding the CD diet, the liver developed a severe fatty change which involved the cells of the entire liver lobule. Although the fatty change persisted for the duration of the experiments, during feeding of the CD diet small areas of hepatocytes showing little or no fatty change began to appear. After 2 weeks on the same diet, the liver of rats in Expts 1, 3 and 5 showed distinct, nodular foci of hepatocytes which, on this basis, could be readily distinguished from the surrounding fatty parenchyma (Fig. 1). The hepatocytes in the foci were arranged irregularly without a distinct sinusoidal pattern and in plates more than one cell thick, had slightly basophilic cytoplasm, contained an occasional small droplet of fat and displayed frequent mitosis (Fig. 2). Foci of hepatocytes identical to these also developed in the liver of the rats in Expts 2 and 4 after 4 weeks of feeding the CD diet. In general, the number of foci increased over the period of feeding the diet. However, gross alterations of the surface of the liver were present in none of the animals. In rats administered 15 mg DEN/kg after PH (Expt 4) and killed after 8 weeks on the CD diet, a slight periportal fibrosis was present in addition to many foci of basophilic, fat-free hepatocytes. Oval cells and/or intermediate cells were frequently seen scattered throughout the parenchyma and often at the periphery of the foci of basophilic, fat-free hepatocytes (Fig. 3). In Expts 1, 2, 4 and 5, the liver of rats on the CS diet showed a well preserved lobular architecture and fatty change, necrosis, inflammation or fibrosis were never seen. However, foci of basophilic hepatocytes similar to those observed in the liver of rats fed the CD diet were observed in rats fed the CS diet in Exp. 3.

*Histochemistry*

After histochemical staining of liver sections, foci of $GGT^+$ hepatocytes were readily seen in rats fed the CD diet but, in those fed the CS diet, only in Expt 3 (Fig. 4). In rats fed the CD diet, the

FIG. 4.—Oval cells and/or intermediate cells (arrows) at the periphery of a focus of altered, basophilic hepatocytes (lower half). Liver section of a rat treated as indicated in Fig. 2. H. and E. ×540.

FIG. 5.—Foci of $\gamma$-glutamyltranspeptidase-positive hepatocytes (arrows) in the liver of a rat treated as in Fig. 2. Cryostat section × 30.

hepatocytes in the foci, in addition to the GGT positivity, also showed little or no fatty change. The latter property indicates that the GGT$^+$ foci correspond to the foci of basophilic hepatocytes observed in the HE-stained sections. Data on the quantitation and diameter of the GGT$^+$ foci are presented in Table I. Significantly more foci developed when the single, initiator dose of DEN was administered to the rats 18 h after PH (Expts 3 and 4) than when it was administered to intact rats (Expts 1 and 2). Actually few foci developed in the liver of rats fed the CS diet only in Expt 3, in which the animals received 30 mg DEN/kg after PH. After administration of 30 mg DEN/kg to intact rats, performance of PH 1 week after the rats had been on the CD diet slightly increased the number of foci formed (Expt 5 *vs* Expt 2). In general, the number as well as the size of the foci increased with increasing time on the CD diet. Bile-duct and ductular cells, and oval and/or intermediate cells were also intensely GGT$^+$. No foci of GGT$^+$ hepatocytes have been seen so far to develop in the liver of rats subjected to no other experimental manipulation than feeding either the CS or the CD diet for up to 6 months.

*Serum enzymes and plasma and liver AFP*

SGOT and plasma GGT and AFP levels were determined in some of the experiments (Table II). SGOT levels were consistently higher in rats on the CD diet than on

TABLE II.—*Levels of serum and plasma enzymes and of plasma AFP*

| Diet | Weeks on diet | No. rats | SGOT* | GGT* | AFP† |
|---|---|---|---|---|---|
| Expt 1 (IR—150 mg DEN) | | | | | |
| CS | 2 | 3 | 54 ± 4 | 0·3 ± 0·3 | 0·10 ± 0·01 |
| CD | 2 | 3 | 353 ± 5 | 3·3 ± 1·7 | 0·22 ± 0·02 |
| CS | 3 | 3 | 49 ± 4 | 1·7 ± 1·2 | 0·08 ± 0·01 |
| CD | 3 | 3 | 279 ± 20 | 16·3 ± 12·3 | 0·18 ± 0·01 |
| Expt 4 (PH—15 mg DEN) | | | | | |
| CS | 4 | 3 | 55 ± 4 | 0·7 ± 0·7 | 0·11 ± 0·03 |
| CD | 4 | 3 | 197 ± 56 | 6·7 ± 3·2 | 0·14 ± 0·04 |
| CS | 8 | 3 | 51 ± 3 | 0·7 ± 0·7 | 0·06 ± 0·01 |
| CD | 8 | 3 | 98 ± 8 | 3·0 ± 1·2 | 0·11 ± 0·01 |

Each value represents the mean ± s.e.
* International units/ml.
† $\mu$g/ml.

the CS diet. Levels of plasma GGT showed on the whole a good correlation with the number of $GGT^+$ foci in the livers. Levels of plasma AFP were consistently higher in rats on the CD diet than in those on the CS diet, but there was no correlation between plasma AFP levels and number of foci in the liver. After immunofluorescence staining of liver sections, most $AFP^+$ cells were oval and/or intermediate cells infiltrating the parenchyma and at the periphery of the $GGT^+$ foci, and an occasional ductular cell. Foci of basophilic, $GGT^+$ hepatocytes were consistently $AFP^-$. Albumin positivity was displayed by some oval and/or intermediate cells, by some hepatocytes in the $GGT^+$ foci, as well as by some surrounding hepatocytes.

### DISCUSSION

The results of the present study show clearly that, after administration to rats of a single initiator dose of a chemical hepatocarcinogen, feeding the animals with a CD diet efficiently and reproducibly promotes in a relatively short period of time the proliferation of initiated liver cells, and their evolution to foci of altered hepatocytes. In Solt & Farber's procedure (1976), such a promoting action is achieved *via* 2 effects, a growth stimulus for initiated cells provided by PH or other mitogenic agents, and selective inhibition of the proliferation of uninitiated cells by exposing the animals to AAF. It is evident, therefore, that similar effects can also be induced by a diet devoid of choline without any further manipulation of the animals. Indeed, in another study (Shinozuka *et al.*, 1979) a CD diet containing AAF was found to have as strong a promoting action as PH and exposure to AAF; that is, the CD diet could effectively replace PH in the original procedure of Solt and Farber. The results of Expts 2 and 5 of the present paper show that the diet can also replace AAF, but less efficiently, since the number of foci/$cm^2$ of liver section in Expt 5 was only slightly more than that in Expt 2 (Table I). This fact may explain why 2–4 weeks on the CD diet are required to induce foci of altered hepatocytes, in contrast to the 7–10 days required in Solt and Farber's procedure.

Unlike the CD diet, the CS diet has no promoting action. Indeed, no foci, or only a minimum number of foci of altered hepatocytes were seen in the present study in rats injected with a single dose of DEN, and then fed the CS diet for various periods of time. Irrespective of the diet fed, the number of foci induced in the liver was greater when an initiator dose of 30 mg DEN/kg body weight was administered 18 h after PH rather than to intact rats (Table I, Expts 2 and 3). This finding is consistent with that of other studies (Scherer & Emmelot, 1976) and may be explained on the basis of a recent report by Cayama *et al.* (1978) that the number of liver cells initiated by a single dose of a chemical carcinogen is maximal when the chemical is administered 18 h after PH. An increase in number as well as in size of the induced foci was found to occur with increasing duration on the diets. The increase in number may be due either to an increasing fraction of initiated cells being promoted, with time, to proliferate and evolve or, more probably, to more foci attaining the minimum scoring diameter of 125 $\mu$m. The increase in diameter is undoubtedly due to cell proliferation. Indeed, in HE-stained sections, hepatocytes in mitosis were frequently seen in the foci (Fig. 2). Despite no histological evidence of hepatocyte necrosis, SGOT levels in rats fed the CD diet were higher than in rats fed the CS diet (Table II). A low rate of liver cell death could represent one possible mechanism underlying the promoting action of the diet (Shinozuka *et al.*, 1979). On the whole, levels of plasma GGT showed good correlation with the number of foci of $GGT^+$ hepatocytes in the livers. Therefore the serum enzyme levels could be used to monitor the emergence, number and further evolution of the foci, were it not that GGT is also present in, and possibly secreted by, bile ductular and duct cells and oval and/or intermediate cells. On the other hand, no correlation was found to exist between serum AFP levels and number of foci in the liver and, by immunofluorescence staining, the cells in the foci were found to be consistently $AFP^-$. The sefindings are consistent with those made and discussed in another study (Shinozuka *et al.*, 1979).

In the present experiments, the foci of altered hepatocytes that developed in the liver of rats on the CD diet had properties very similar to those described by Solt & Farber (1976) and Solt *et al.* (1977) for the foci induced with their procedure, such as arrangement of the hepatocytes in plates more than one cell thick, basophilia of the cytoplasm, GGT positivity, and a high mitotic rate. Administration of a single initiator dose of 30 mg DEN/kg 18 h after PH, followed by feeding the CD diet for 4 weeks, led to the formation of the largest number of foci, 27/cm$^2$ of liver section, or $2{\cdot}5 \times 10^4$/liver (Table I, Expt 3). These numbers are certainly adequate for carrying out kinetic and other types of study on the origin, development and further evolution of the foci. Large numbers might be induced, if needed, by administration of a larger dose of DEN, since it has been shown that there is a direct relationship between the size of the initial dose of DEN and the number of foci formed in the liver (Solt & Farber, 1976; Shinozuka *et al.*, 1979). It is evident, therefore, that administration of a single initiator dose of a chemical carcinogen, 18 h after PH, followed by feeding laboratory chow for 1 week and the CD diet for varying lengths of time (depending mostly upon the size of the initiator dose and the number of foci/liver required) constitutes a new and efficient procedure for the induction of foci of altered hepatocytes in rat liver. The new procedure appears to have at least one major advantage over the original procedure of Solt and Farber, namely, that it requires the use of a simpler selection pressure, one avoiding the need for application of a strong mitogenic stimulus such as PH or other mitogenic chemicals and, more importantly, the use of AAF, a second chemical hepatocarcinogen. Another advantage, of secondary importance, is the availability of a new marker for the identification of the foci, namely, the absolute or relative absence of fatty change. In the experiments reported in this paper, as well as in those described elsewhere (Shinozuka *et al.*, 1979), identification of foci of basophilic hepatocytes in HE-stained sections of livers from rats on the CD diet was consistently made easy by the fact that the basophilic hepatocytes had no or very little fatty change, in contrast to the surrounding hepatocytes (Fig. 1). The same property was shown in histochemically stained sections by the hepatocytes of the $GGT^+$ foci, indicating the identity of the foci. The new procedure, used alone or in conjunction with that of Solt and Farber, may prove to be a valuable, alternative or additional tool in studies concerned with the analysis of the early steps and cellular events of chemical hepatocarcinogenesis, and the mechanism(s) underlying promotion of liver carcinogenesis.

This study was supported by Research Grants CA 23499 and CA 21230 from the National Cancer Institute and a grant from the Samuel and Emma Winters Foundation. We wish to express our thanks to Ms E. Jahnke, Maggie Nefores and Ruth Alexander for their expert technical assistance, and to Ms C. Channing for typing the manuscript.

### REFERENCES

CAYAMA, E., SARMA, D. S. R. & FARBER, E. (1978) Initiation of chemical carcinogenesis as a stepwise process requiring cell proliferation. *Nature*, **275**, 60.

FARBER, E. (1976) On the pathogenesis of experimental hepatocellular carcinoma. In *Hepatocellular Carcinoma*. Ed. K. Okuda & R. L. Peters. New York: John Wiley. p. 3.

HIGGINS, G. M. & ANDERSON, R. M. (1931) Experimental pathology of the liver. I. Restoration of the liver of the white rat following partial surgical removal. *Arch. Pathol.*, **12**, 186.

LAISHES, B. A. & FARBEP, E. (1978) Transfer of viable putative preneoplastic hepatocytes to the livers of syngeneic host rats. *J. Natl Cancer Inst.*, **61**, 507.

LOMBARDI, B. (1971) Effects of choline deficiency on rat hepatocytes. *Fed. Proc.*, **30**, 139.

LOMBARDI, B. & SHINOZUKA, H. (1979) Enhancement of 2-acetylaminofluorene liver carcinogenesis in rats fed a choline-devoid diet. *Int. J. Cancer*, **23,** 565.

Peraino, C., Fry, R. J. M., Staffeldt, E. & Kisieleski, W. E. (1973) Effects of varying the exposure to phenobarbital on its enhancement of 2-acetylaminofluorene-induced hepatic tumorigenesis in the rat. *Cancer Res.*, **33,** 2701.

Pitot, H. C., Barsness, L., Goldsworthy, T. & Kitagawa, T. (1978) Biochemical characterization of stages of hepatocarcinogenesis after a single dose of diethylnitrosamine. *Nature*, **271,** 456.

Rutenberg, A. M., Kim, H., Fischbein, J. W., Hankers, J. S., Wasserkrug, H. L. & Seligman, A. M. (1969) Histochemical and ultrastructural demonstration of $\gamma$-glutamyltranspeptidase activity. *J. Histochem. Cytochem.*, **17,** 517.

Scherer, E. & Emmelot, P. (1975) Foci of altered liver cells induced by a single dose of diethylnitrosamine and partial hepatectomy: their contribution to hepatocarcinogenesis in the rat. *Eur. J. Cancer*, **11,** 145.

Scherer, E. & Emmelot, P. (1976) Kinetics of induction and growth of enzyme-deficient islands involved in hepatocarcinogenesis. *Cancer Res.*, **36,** 2544.

Scherer, E., Hoffman, M., Emmelot, P. & Friedrich-Freksa, H. (1972) Quantitative study on foci of altered liver cells induced in the rat by a single dose of diethylnitrosamine and partial hepatectomy. *J. Natl Cancer Inst.*, **49,** 93.

Sell, S. (1978) Distribution of $\alpha_1$-fetoprotein and albumin containing cells in the livers of Fisher rats fed four cycles of N-2 fluorenylacetamide. *Cancer Res.*, **38,** 3107.

Sell, S. & Gord, D. (1973) Rat alpha-fetoprotein. III. Refinement of radioimmunoassay for detection of 1 ng rat $\alpha_1$F. *Immunochemistry*, **10,** 439.

Shinozuka, H., Katyal, S. L. & Lombardi, B. (1978*a*) Azaserine carcinogenesis: organ susceptibility in rats fed a diet devoid of choline. *Int. J. Cancer*, **22,** 36.

Shinozuka, H., Lombardi, B., Sell, S. & Iammarino, R. M. (1978*b*) Enhancement of ethionine liver carcinogenesis in rats fed a choline-devoid diet. *J. Natl Cancer Inst.*, **61,** 813.

Shinozuka, H., Lombardi, B., Sell, S. & Iammarino, R. M. (1978*c*) Early histological and functional alterations of ethionine liver carcinogenesis in rats fed a choline-deficient diet. *Cancer Res.*, **38,** 1902.

Shinozuka, H., Sells, M. A., Katyal, S. L., Sell, S. & Lombardi, B. (1979) Effects of a choline-devoid diet on the emergence of $\gamma$-glutamyltranspeptidase-positive foci in the liver of carcinogen-treated rats. *Cancer Res.*, **39,** 2515.

Solt, D. B. & Farber, E. (1976) A new principle for the sequential analysis of chemical carcinogenesis including a quantitative assay for initiation in liver. *Nature*, **263,** 701.

Solt, D. B., Medline, A. & Farber, E. (1977) Rapid emergence of carcinogen-induced hyperplastic lesions in a new model for the sequential analysis of liver carcinogenesis. *Am. J. Pathol.*, **88,** 595.


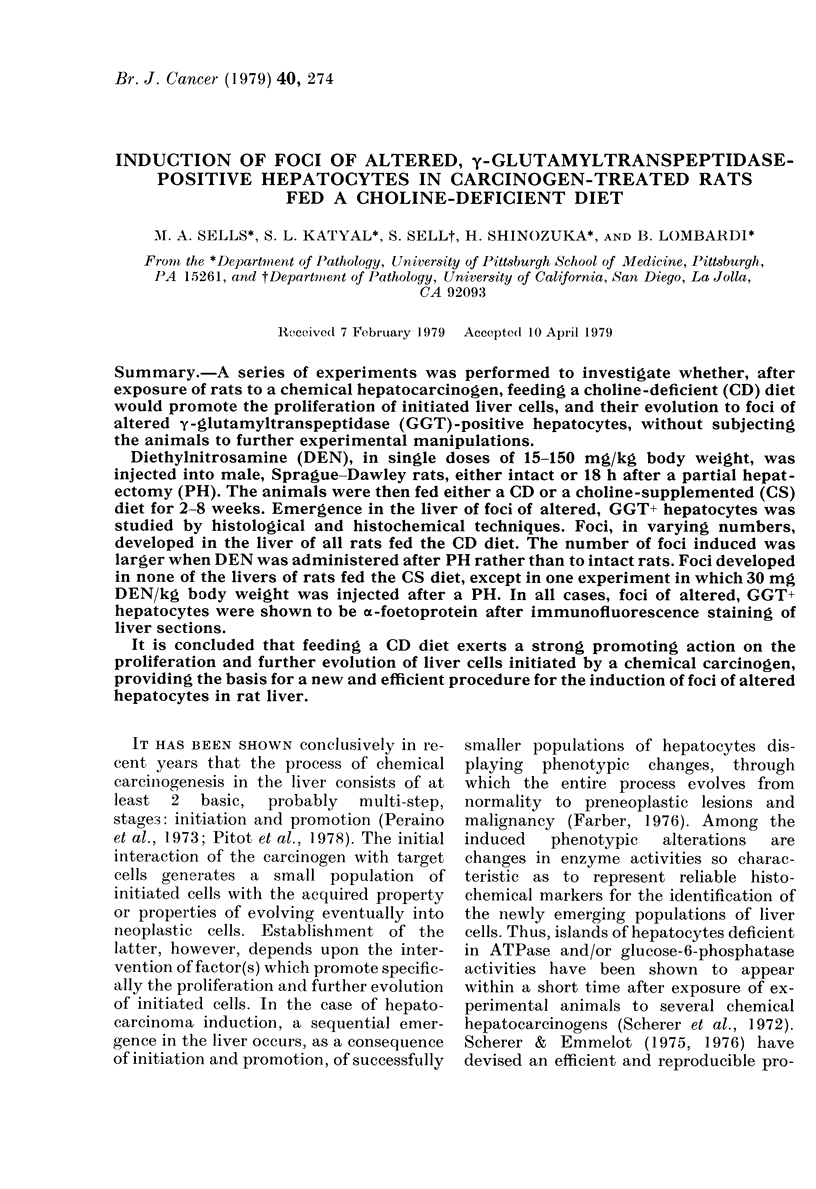

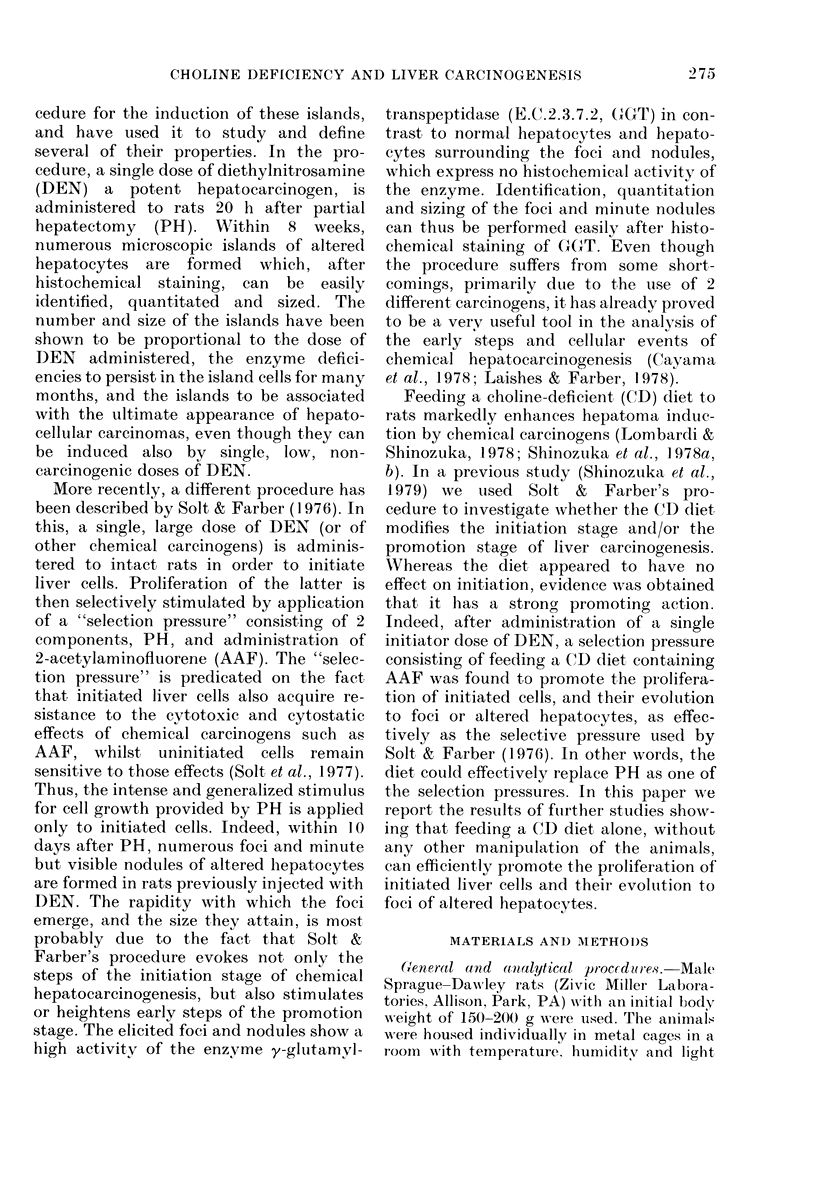

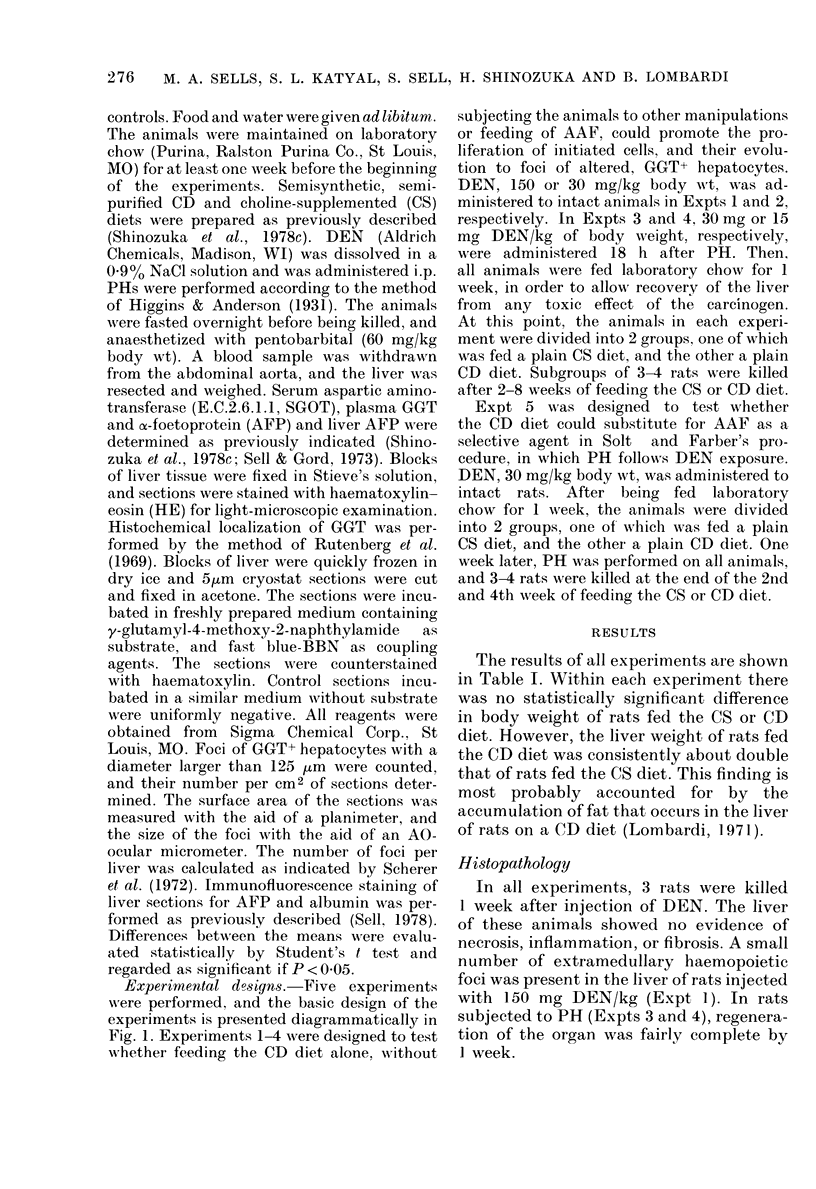

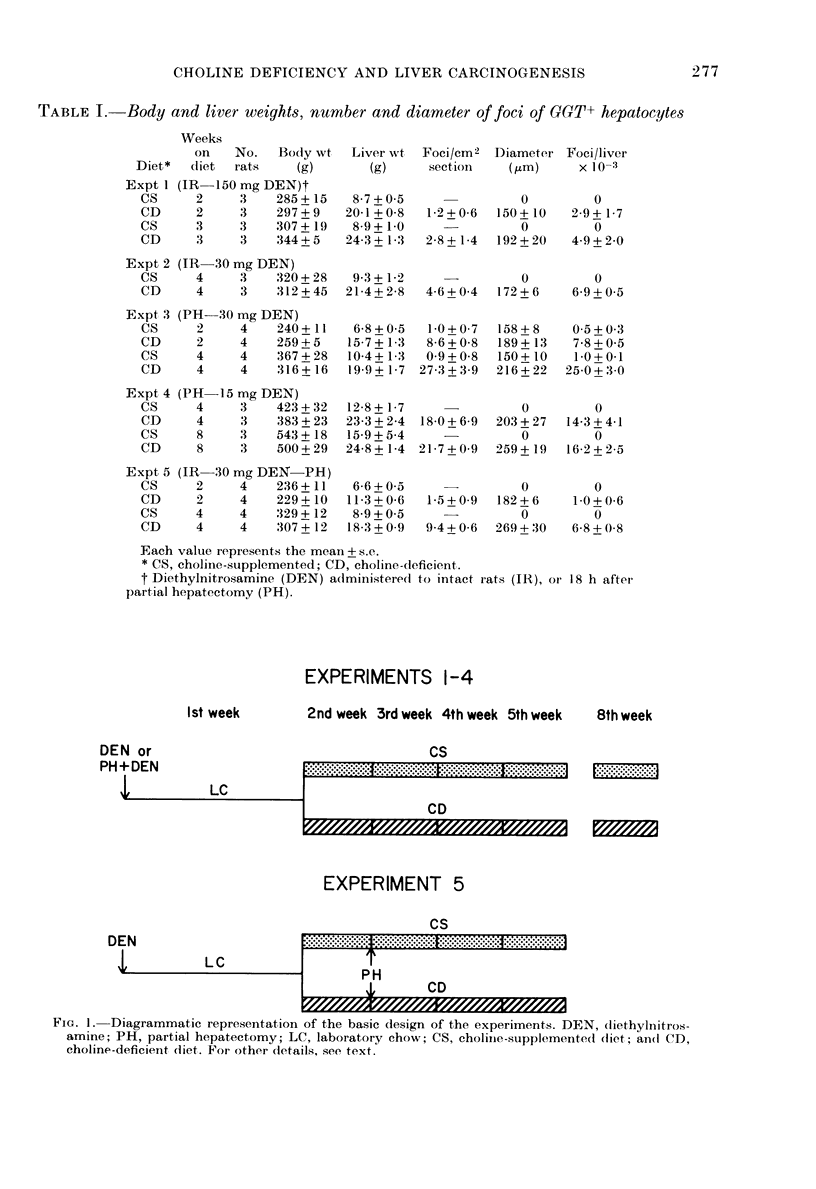

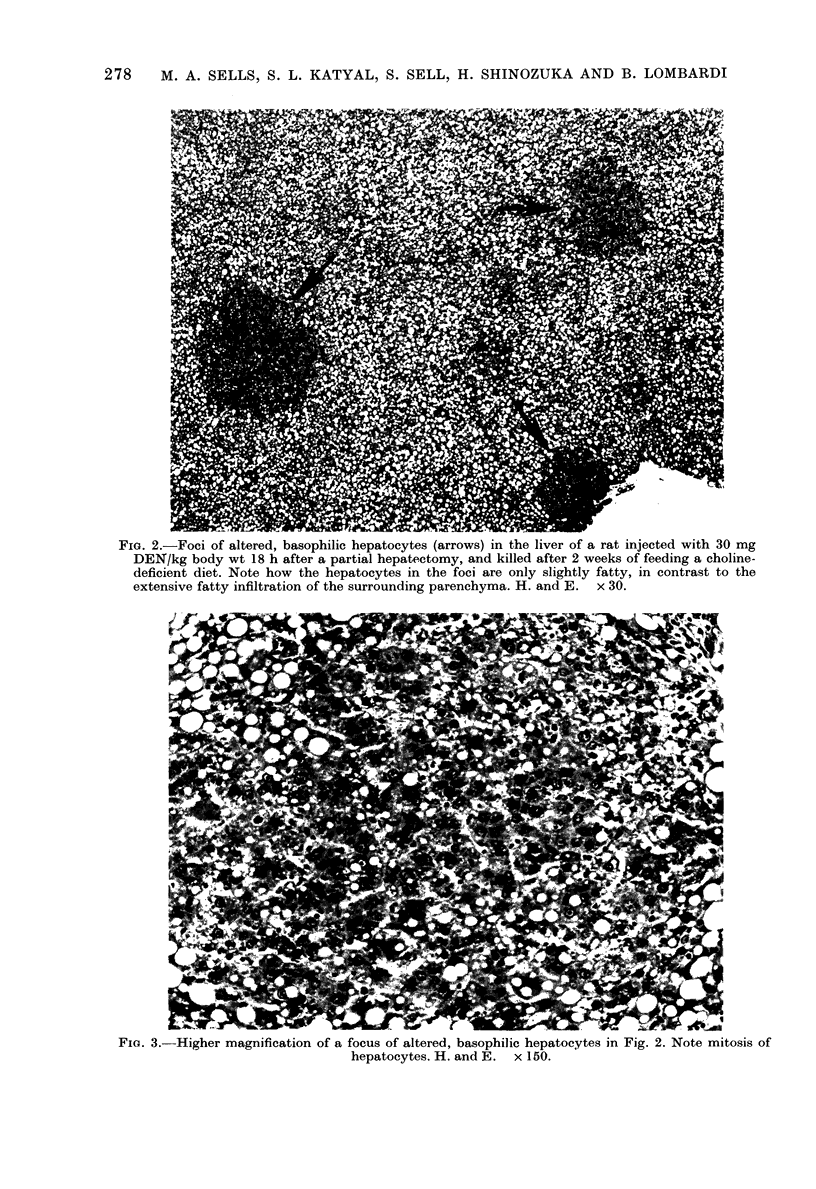

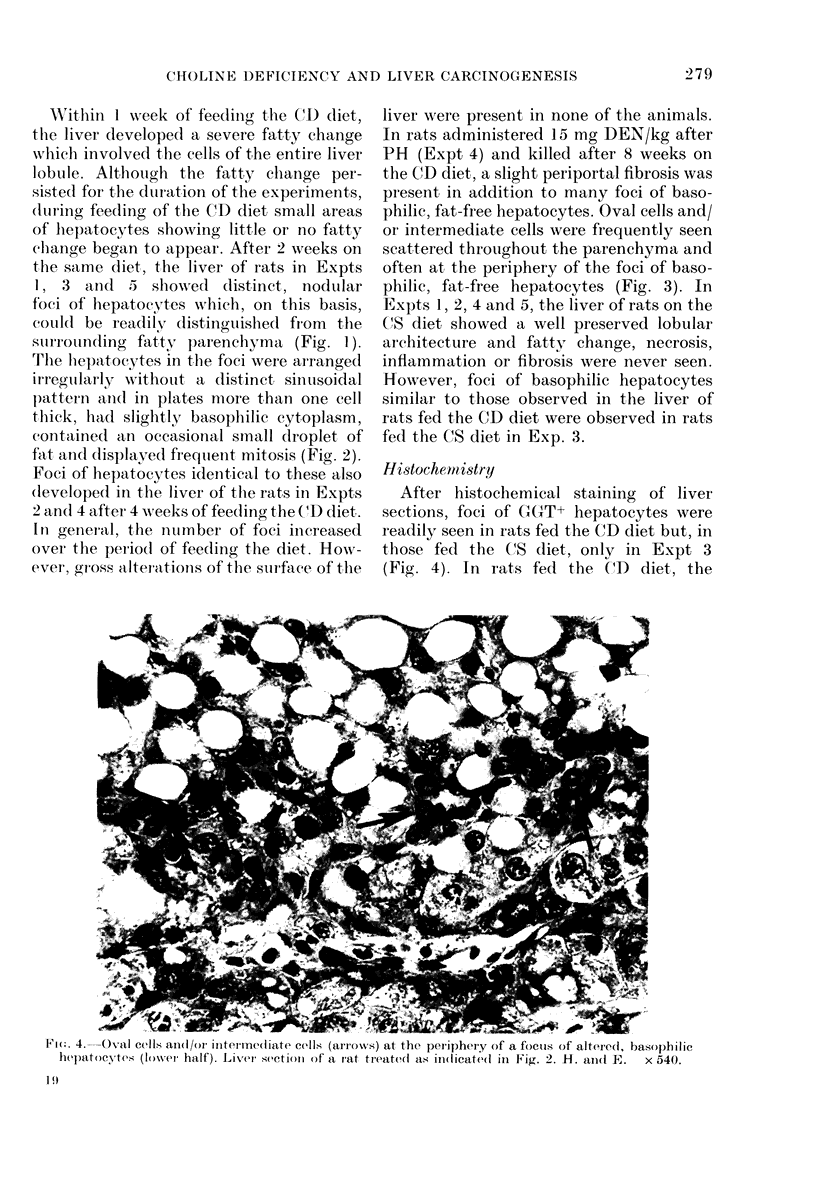

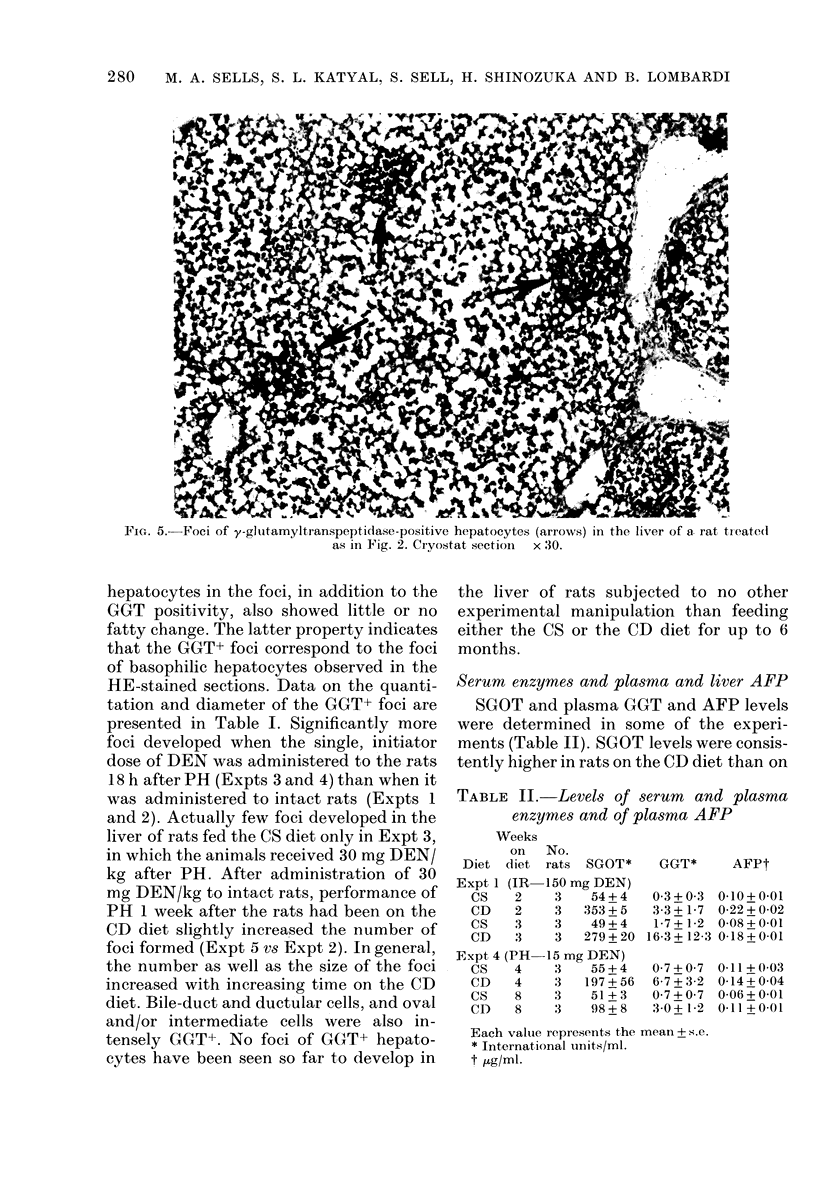

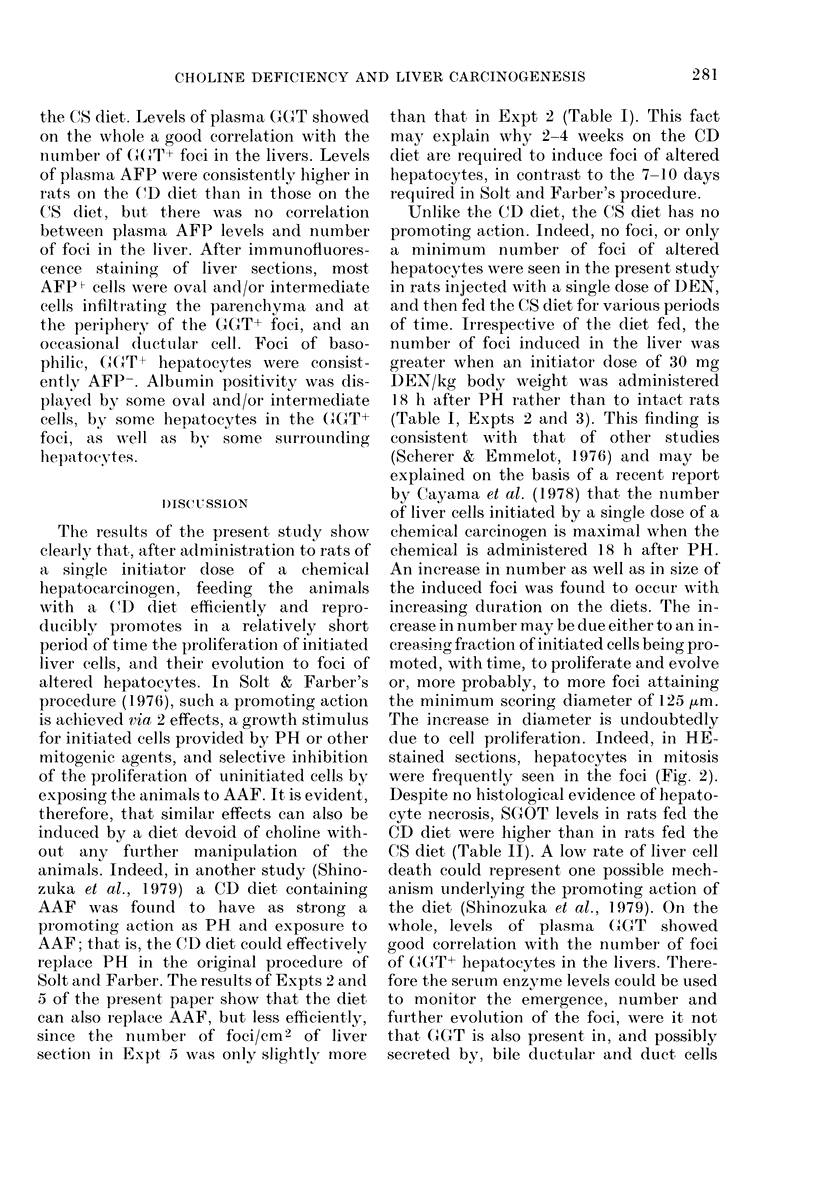

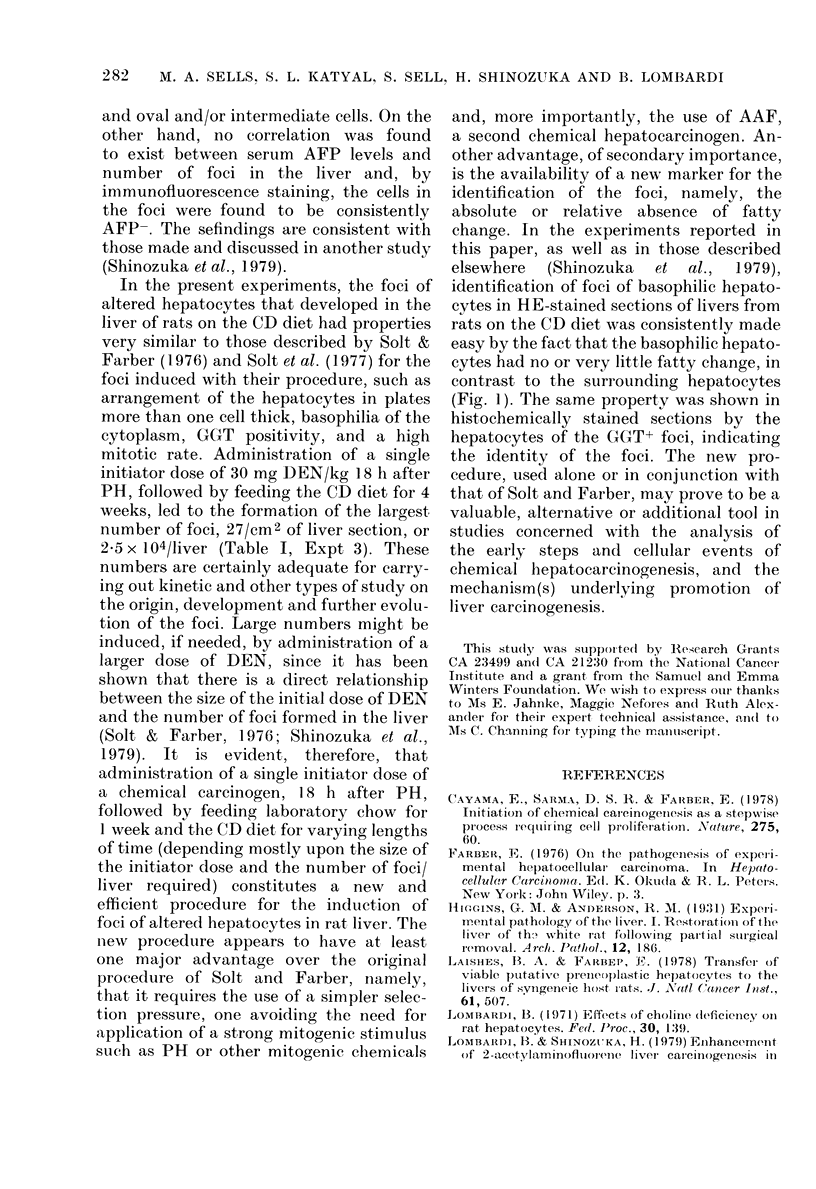

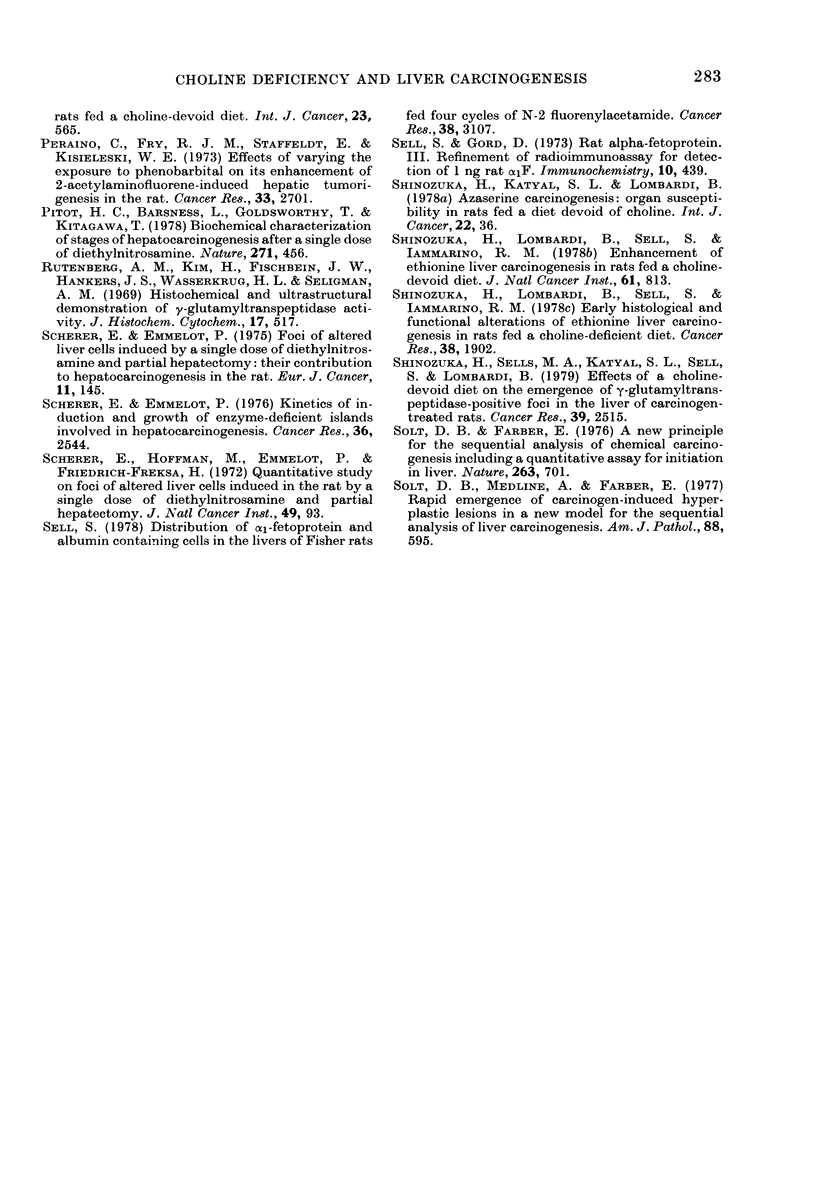


## References

[OCR_00971] Laishes B. A., Farber E. (1978). Transfer of viable putative preneoplastic hepatocytes to the livers of syngeneic host rats.. J Natl Cancer Inst.

[OCR_00977] Lombardi B. (1971). Effects of choline deficiency on rat hepatocytes.. Fed Proc.

[OCR_00990] Peraino C., Fry R. J., Staffeldt E., Kisieleski W. E. (1973). Effects of varying the exposure to phenobarbital on its enhancement of 2-acetylaminofluorene-induced hepatic tumorigenesis in the rat.. Cancer Res.

[OCR_00997] Pitot H. C., Barsness L., Goldsworthy T., Kitagawa T. (1978). Biochemical characterisation of stages of hepatocarcinogenesis after a single dose of diethylnitrosamine.. Nature.

[OCR_01003] Rutenburg A. M., Kim H., Fischbein J. W., Hanker J. S., Wasserkrug H. L., Seligman A. M. (1969). Histochemical and ultrastructural demonstration of gamma-glutamyl transpeptidase activity.. J Histochem Cytochem.

[OCR_01010] Scherer E., Emmelot P. (1975). Foci of altered liver cells induced by a single dose of diethylnitrosamine and partial hepatectomy: their contribution to hepatocarcinogenesis in the rat.. Eur J Cancer.

[OCR_01017] Scherer E., Emmelot P. (1976). Kinetics of induction and growth of enzyme-deficient islands involved in hepatocarcinogenesis.. Cancer Res.

[OCR_01023] Scherer E., Hoffmann M., Emmelot P., Friedrich-Freksa M. (1972). Quantitative study on foci of altered liver cells induced in the rat by a single dose of diethylnitrosamine and partial hepatectomy.. J Natl Cancer Inst.

[OCR_01030] Sell S. (1978). Distribution of alpha-fetoprotein- and albumin-containing cells in the livers of Fischer rats fed four cycles of N-2-fluorenylacetamide.. Cancer Res.

[OCR_01037] Sell S., Gord D. (1973). Rat alpha-fetoprotein. 8. Refinement of radioimmunoassay for detection of 1 ng rat alpha 1F.. Immunochemistry.

[OCR_01042] Shinozuka H., Katyal S. L., Lombardi B. (1978). Azaserine carcinogenesis: organ susceptibility change in rats fed a diet devoid of choline.. Int J Cancer.

[OCR_01048] Shinozuka H., Lombardi B., Sell S., Iammarino R. M. (1978). Enhancement of DL-ethionine-induced liver carcinogenesis in rats fed a choline-devoid diet.. J Natl Cancer Inst.

[OCR_01061] Shinozuka H., Sells M. A., Katyal S. L., Sell S., Lombardi B. (1979). Effects of a choline-devoid diet on the emergence of gamma-glutamyltranspeptidase-positive foci in the liver of carcinogen-treated rats.. Cancer Res.

[OCR_01074] Solt D. B., Medline A., Farber E. (1977). Rapid emergence of carcinogen-induced hyperplastic lesions in a new model for the sequential analysis of liver carcinogenesis.. Am J Pathol.

